# Singlet Oxygen in Plants: Mechanistic Insights into Production, Oxidative Damage, and Stress-Response Signaling

**DOI:** 10.3390/antiox15080952

**Published:** 2026-07-30

**Authors:** Zhonghua Qiu, Jiayu Wang

**Affiliations:** Key Laboratory of Rice Biology & Genetic Breeding in Northeast China, Ministry of Agriculture and Rural Areas, Rice Research Institute, Shenyang Agricultural University, Shenyang 110866, China; 2023200088@stu.syau.edu.cn

**Keywords:** photodamage, photoinhibition, retrograde signaling, singlet oxygen

## Abstract

Singlet oxygen (^1^O_2_), a highly reactive oxygen species generated in illuminated chloroplasts, is a major contributor to photooxidative damage in plants, particularly under high-light stress. This review summarizes the major sites of ^1^O_2_ production, its damage mechanisms, and the antioxidant defense and scavenging strategies that limit ^1^O_2_ accumulation. Particular emphasis is placed on recent advances in ^1^O_2_-mediated signal transduction. EX1 and β-carotene-derived oxidation products have been proposed to act as key components in ^1^O_2_ perception and signaling, mediating signal transmission from distinct subchloroplast locations and contributing to a spatially coordinated ^1^O_2_ signaling network. Plants counteract ^1^O_2_-induced damage through a multilayered defense–perception–signal transduction system. The functional partitioning of ^1^O_2_ signaling between grana margins and grana cores, together with the coordinated action of multiple signaling molecules, provides important insights into the mechanisms of plant photoprotection and offers potential strategies for improving crop tolerance to photooxidative stress.

## 1. Introduction

Chloroplasts are the primary sites for photosynthesis in plants and also serve as central hubs for perceiving and transmitting signals triggered by biotic and abiotic stresses [1,2,3,4]. When studying plant photosynthesis, it involves not only a series of complex processes such as light energy capture and electron transfer but also requires consideration of signal transduction between plastids and the nucleus, particularly plastid-to-nucleus retrograde signaling (RS) [5,6]. Singlet oxygen (^1^O_2_ or SO) is a type of reactive oxygen species (ROS) produced by plants during photodamage. It not only exerts a significant impact on the regulation of photosynthesis but also plays a crucial role in plants’ response to environmental stresses. In-depth research on the regulatory mechanisms of ^1^O_2_ in plants is of great significance for studies related to plant resistance to adverse stresses [7,8]. This article summarizes the production, damage mechanisms, and response strategies of ^1^O_2_ in plants, providing a theoretical basis for future in-depth research.

## 2. Production of Singlet Oxygen

Biotic and abiotic stresses in the environment usually exert harmful effects on plants. As autotrophs, plants rely on light energy from the environment to drive photosynthesis and a series of physiological processes. Excessive light can reduce the photosynthetic rate, a phenomenon known as photoinhibition [9,10,11]. Beyond excessive light, photoinhibition can also occur under normal light conditions when stresses such as drought and low temperature are present. Under normal growth conditions, the light energy absorbed by plants not only meets the needs of photosynthesis but also the excess energy is dissipated in the form of chlorophyll fluorescence or non-photochemical quenching (NPQ) [12,13,14].

However, under photoinhibition, the presence of excess light energy prevents effective quenching, which disrupts normal photosynthesis, impairs plant growth and development, and may even lead to plant death [15]. Under photoinhibitory conditions, the absorbed energy cannot be consumed in a timely manner, causing damage to photosynthetic apparatus and generating reactive oxygen species (ROS), such as ^1^O_2_ and superoxide anion radicals (O_2_^−^) [16,17]. A moderate level of ROS production can regulate plant gene expression and physiological status, enabling plants to respond to changes in the external environment. When the production rate of ROS exceeds the scavenging capacity of the antioxidant network, irreversible damage to lipids, proteins, pigments, and nucleic acids may occur [18]. Among these ROS, ^1^O_2_ plays a crucial role in photosynthesis. Since ^1^O_2_ is mainly produced during photosynthesis and exhibits strong oxidizing properties towards photosynthesis-related proteins, it is considered a major factor contributing to photodamage.

### 2.1. Singlet Oxygen Production by Light-Harvesting Complexes

Light-harvesting complexes (LHCs) are pigment–protein complexes embedded in the thylakoid membrane that absorb light and transfer excitation energy to photosynthetic reaction centers. Light-harvesting complex II (LHCII) is the major antenna complex of photosystem II (PSII). Because of its abundant chlorophyll content and close association with the PSII reaction center, LHCII, together with the PSII reaction center, is considered a major site of ^1^O_2_ production in the photosynthetic system [18,19]. Compared with PSII, the photosystem I (PSI) complex is relatively stable but also produces small amounts of ^1^O_2_ [20]. LHCII is a complex formed by light-harvesting antenna proteins (Lhcb1-Lhcb6) and chlorophyll; the absence of LHCII exposes the PSII reaction center to light [21,22,23]. Chlorophyll a oxygenase(CAO), which converts chlorophyll a to chlorophyll b through oxidation [24]. The *Arabidopsis thaliana ch1* mutant, due to the inactivation of AtCAO, exhibits a lack of chlorophyll b and LHCII. Under high light, *ch1* shows extremely high sensitivity and produces large amounts of ^1^O_2_ [25,26].

**Figure 1:**
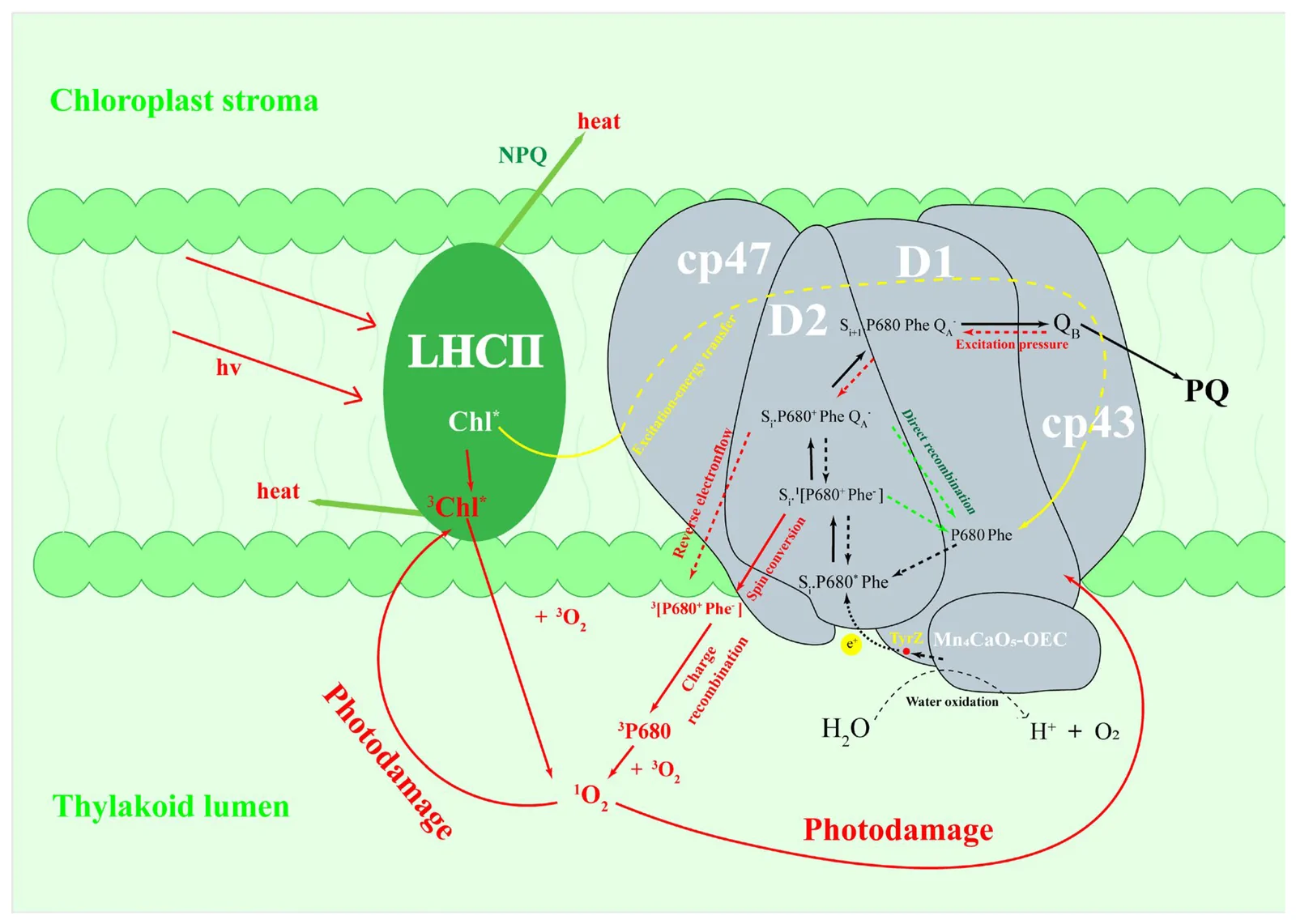
Schematic model of ^1^O_2_ production and photodamage in thylakoid membrane LHCII and PSII complexes. Excess light energy absorbed by LHCII is either thermally dissipated through NPQ or converted to triplet chlorophyll (^3^Chl*) that generates ^1^O_2_. High excitation pressure induces multiple charge recombination reactions in the PSII reaction center to form triplet P680 (^3^P680), which reacts with triplet oxygen to produce ^1^O_2_. The accumulated ^1^O_2_ leads to photodamage of photosynthetic apparatus. Normal photosynthetic electron transport coupled with water oxidation by the Mn_4_CaO_5_-OEC is also shown.

The PSII reaction center is mainly centered on a heterodimer formed by the D1 protein (PsbA) and D2 protein (PsbD). Tightly bound around this heterodimer core are proteins such as the inner peripheral light-harvesting antenna proteins CP43 and CP47, as well as pigments including chlorophyll a and pheophytin [27,28]. In LHCII and the PSII reaction center, chlorophyll converts to the singlet excited state (^1^Chl*) after absorbing light energy. Under conditions in which excitation energy cannot be efficiently utilized in photochemical reactions or dissipated as heat, ^1^Chl* is more likely to undergo intersystem crossing (ISC) and generates triplet chlorophyll (^3^Chl*) [29,30,31]. Compared with ^1^Chl* (with a lifetime of approximately 10^−8^ s), ^3^Chl* has a longer lifetime (approximately 10^−3^ s), which allows sufficient time for ^3^Chl* to react with ground-state oxygen and produce ^1^O_2_ [7,32]. In addition to abnormal energy transfer, abnormal electron transfer also leads to ^1^O_2_ production. Under normal conditions, after PSII absorbs light energy, P680 converts to the excited state (P680*). Subsequently, P680* transfers electrons to pheophytin (Pheo), converting Pheo to the reduced state (Pheo^−^) while P680 itself converts to the oxidized state (P680^+^). Electrons are then transferred via plastoquinone (PQ), and P680^+^ reverts to the ground state by accepting electrons produced from water splitting by the oxygen-evolving complex (OEC), thus completing the normal electron cycle [19,33]. When PSII electron transfer is impaired, for example by D1 protein damage—which compromises the PSII reaction center and may lead to over-reduction of the PQ pool—or by insufficient availability of downstream electron acceptors, reduced electron carriers such as Pheo^−^ may accumulate and transfer electrons to O_2_, generating O_2_·^−^. In addition, impaired charge recombination under these conditions may promote the formation of ^3^Chl* and consequently enhance ^1^O_2_ generation through energy transfer to O_2_ (Figure 1) [34,35]. Under conditions that impair PSII stability or electron transfer, the probability of abnormal charge recombination increases, which can enhance ^1^O_2_ formation [36,37,38].

### 2.2. Chlorophyll and Other Photosensitizers Involved in Singlet Oxygen Production

Many endogenous substances in plants can act as photosensitizers. After absorbing light energy of specific wavelengths, such substances are excited and further generate ^1^O_2_ through energy transfer. Among the known endogenous photosensitizers, tetrapyrrole intermediates, particularly porphyrins and chlorophyll precursors, play a prominent role in plant ^1^O_2_ production [39,40]. The accumulation of porphyrin intermediates provides one example of how disruption of tetrapyrrole metabolism promotes ^1^O_2_ formation [41].

*AtFC2* encodes one of the two ferrochelatases in *Arabidopsis thaliana* and is mainly responsible for inserting Fe^2+^ into protoporphyrin IX (Proto IX) to produce heme. In the *fc2* mutant, the accumulated Proto IX acts as a photosensitizer and generates ^1^O_2_, causing damage to chloroplasts. The damaged chloroplasts are degraded via ubiquitination mediated by the PUB4 ubiquitin ligase [42]. This example illustrates that the accumulation of porphyrin intermediates can directly connect defects in tetrapyrrole metabolism with chloroplast photooxidative damage. Chlorophyll biosynthetic intermediates provide another well-established source of photosensitizer-dependent ^1^O_2_. *FLU* encodes a negative regulator of tetrapyrrole biosynthesis. Under dark conditions, protochlorophyllide (Pchlide) accumulates extensively in the *flu* mutant (which lacks this regulator). When *flu* mutants acclimated to dark conditions are transferred to light, the burst of ^1^O_2_ causes photobleaching in seedlings and inhibits seedling growth [43]; however, inactivation of the EXECUTER1 (EX1) can rescue the phenotype of the *flu* mutant [44,45]. This finding indicates that EX1 functions primarily downstream of ^1^O_2_ production, linking ^1^O_2_ accumulation to chloroplast-to-nucleus signaling and subsequent stress responses. A similar relationship between Pchlide accumulation and ^1^O_2_ production has been observed in the *porc* mutant [46]. *AtPORC* encodes protochlorophyllide oxidoreductase. Exogenous application of 5-aminolevulinic acid (ALA) leads to the accumulation of Pchlide in the *porc* mutant, producing large amounts of ^1^O_2_ and increasing cell death. In contrast, overexpression of *AtPORC* can reduce the accumulation of protochlorophyllide and the production of ^1^O_2_. Together, these examples demonstrate that disturbances in tetrapyrrole metabolism can promote the accumulation of endogenous photosensitizers, including Proto IX and Pchlide. Under illumination, these intermediates generate ^1^O_2_, which can cause photooxidative damage.

### 2.3. Other Chloroplast Pathways Contributing to Singlet Oxygen Production

Plants can produce ^1^O_2_ in the absence of light. When exposed to various biotic and abiotic stresses in the dark, the singlet oxygen-specific probe Singlet Oxygen Sensor Green (SOSG) detects the rapid production of ^1^O_2_ in the roots of *Arabidopsis thaliana*. Green fluorescence indicates the accumulation of ^1^O_2_ in mitochondria, peroxisomes, and the nucleus [47]. Osmotic stress induced by polyethylene glycol (PEG) causes the rapid appearance of ^1^O_2_ in the epidermal region of *Arabidopsis* root tips, which then dynamically diffuses to other parts. Meanwhile, the use of His (a specific scavenger of ^1^O_2_) can specifically inhibit the accumulation of ^1^O_2_, reducing cell death by 50% and alleviating the inhibition of root elongation caused by PEG [48].

## 3. Singlet Oxygen-Induced Damage to PSII and Thylakoid Membranes

### 3.1. Singlet Oxygen-Induced Damage to Photosystem II

^1^O_2_ is considered a major factor contributing to photodamage in plants. Under high-light conditions, ^1^O_2_ can be generated in photosystem II (PSII) through multiple photochemical pathways, including the formation of excited ^3^Chl* and charge-recombination reactions [49]. Because ^1^O_2_ is produced within or near the photosynthetic apparatus and has a relatively short lifetime and limited diffusion distance, PSII components are particularly susceptible to ^1^O_2_-mediated oxidative damage [49,50]. At the same time, ^1^O_2_ is highly reactive and can oxidize a wide range of cellular components, including aromatic amino acid residues, unsaturated lipids, pigments, and proteins. Oxidative modification of PSII proteins and pigments by ^1^O_2_ can impair the structure and function of the PSII complex, thereby contributing to photoinhibition. In various experimental systems, there is a clear correlation between the formation rate of ^1^O_2_ and the rate of PSII photodamage, supporting a close association between ^1^O_2_ production and impairment of PSII structure and function [51,52].

PSII is a pigment-protein complex composed of over 20 different proteins and pigments, with the D1 and D2 proteins forming its reaction center [53,54]. Unlike the D2 protein, which primarily plays an auxiliary role in charge separation, the D1 protein is directly involved in the charge separation of P680*, making it more susceptible to becoming the main oxidation site of ^1^O_2_ [55]. The replacement of photodamaged D1 protein is an essential step in the PSII repair cycle, Impairment of D1 turnover or PSII reassembly results in the accumulation of inactive or incompletely repaired PSII centers, thereby reducing functional PSII abundance and photosynthetic electron transport efficiency. This limitation subsequently restricts carbon assimilation and photosynthetic product accumulation, and prolonged disruption of PSII repair may contribute to impaired plant growth, biomass production, and yield formation [11,56,57]. Stress-induced damage to D1, together with impairment of the D1 repair cycle, promotes the accumulation of inactive PSII reaction centers, thereby exacerbating sustained photoinhibition and reducing stress tolerance [58]. Precisely because impaired D1 protein affects plant growth, it has become a target for many herbicides [59].

As a type of ROS, ^1^O_2_ has strong oxidizing properties and can react with amino acid residues in adjacent proteins. ROS may induce oxidative post-translational modifications (OPTM) in individual PSII protein subunits, altering their polarity and spatial conformation and inactivating the functional sites of proteins [60]. Reported target amino acids include sulfur-containing cysteine (Cys) and methionine (Met), as well as aromatic amino acids such as tyrosine (Tyr), phenylalanine (Phe), and tryptophan (Trp). Among these, OPTM of Trp is irreversible, so the only way to remove this modification is to degrade the irreversibly oxidized protein [61,62]. In *Arabidopsis thaliana* and *Chlamydomonas*, the side chains of Trp14 and Trp317 in the D1 protein are oxidized to form oxindolylalanine (OIA), N-formylkynurenine (NFK), or kynurenine (KYN), resulting in protein mass shifts [63,64].

^1^O_2_ may damage the D1 protein by oxidizing its Trp residues. Damage and repair of the D1 protein occur simultaneously; when the rate of photodamage exceeds that of repair, the function of the PSII protein complex is impaired [65]. In vitro experiments show that when SDS-solubilized PSII complexes are exposed to ^1^O_2_, no distinct fragments of the D2 protein are detected, while the D1 protein is cleaved into specific fragments [66]. In *Synechococcus* sp. PCC 6803, ^1^O_2_ inhibits de novo protein synthesis, particularly the synthesis of the D1 protein. The accumulation the mRNA of PsbA (which encodes the D1 protein) is not affected by ^1^O_2_ production, but ^1^O_2_ can bind to mRNA of PsbA. Therefore, the main targets of ^1^O_2_ are the translation and elongation steps of mRNA [67]. Additionally, ^1^O_2_ inactivates the chloroplast elongation factor EF-G, which is involved in the reassembly of newly synthesized D1 and D2 proteins during PSII repair [57,68,69].

### 3.2. Singlet Oxygen-Induced Damage to Thylakoid Membranes

The harm of ROS to membranes mainly stems from lipid peroxidation reactions they induce, which trigger a series of chain reactions, ultimately leading to membrane structural destruction and functional loss [70]. Unlike other ROS, ^1^O_2_ exhibits high specificity and slow initiation in damaging biological membranes: it only attacks specific fatty acids containing double bonds, causing a gradual increase in membrane permeability [65,71]. Thylakoid membranes host various protein complexes involved in photosynthetic energy conversion and electron transport, electron carriers, transporters, and abundant pigments. Multiple biological processes occur on thylakoid membranes, such as post-translational regulation and modification of proteins, as well as disassembly and reassembly of protein complexes [72,73]. Moreover, ^1^O_2_ is generated predominantly through energy transfer from triplet chlorophyll, particularly triplet P680 (^3^P680) formed during charge recombination in the PSII reaction center, to molecular oxygen. Owing to its short lifetime and limited diffusion distance, the oxidative activity of ^1^O_2_ is largely confined to its site of generation. Consequently, PSII core proteins, photosynthetic pigments, and neighboring thylakoid membrane lipids are particularly susceptible to ^1^O_2_-mediated oxidative damage, thereby impairing photosynthetic electron transport, destabilizing photosynthetic protein complexes, and ultimately compromising photosynthetic performance.

In the study of *Arabidopsis* SAFEGUARD1, ^1^O_2_ generated from chlorophyll biosynthetic intermediates was associated with a marked decline in protochlorophyllide oxidoreductase (POR), Mg^2+^ chelatase subunit I (CHLI), and Mg^2+^ protoporphyrin IX methyltransferase (CHLM), which are enriched at the grana margins. By contrast, the levels of the grana-core-enriched PSII reaction-center proteins D1 and D2 were not significantly altered under the same experimental conditions. These results indicate that, in this specific tetrapyrrole-associated ^1^O_2_-generating system, proteins located at or near the grana margins are particularly susceptible [74]. Using a multi-well plate screening assay combined with chlorophyll fluorescence imaging, the authors investigated RB-mediated ^1^O_2_ photodamage in isolated thylakoid membranes and intact *Chlorella* cells. In intact cells, increasing the concentration of RB resulted in a progressively greater loss of the maximum quantum yield of PSII (Fv/Fm) after 60 min of illumination, indicating a concentration-dependent increase in PSII photodamage associated with enhanced ^1^O_2_ generation. This effect was particularly pronounced in the presence of lincomycin, which inhibits protein synthesis and limits the repair of photodamaged PSII. In the absence of lincomycin, the RB-induced loss of PSII activity showed an increasing trend with RB concentration and reached statistical significance at 10 μM RB [65].

## 4. Antioxidant Defense Against Singlet Oxygen

To avoid damage from excessive ROS accumulation, plants have evolved a multi-level, highly efficient, and coordinated scavenging system. The protective system is divided into two main categories: enzymatic scavenging systems and non-enzymatic scavenging systems. These two systems work together to maintain the “dynamic balance” of intracellular ROS. Among them, the non-enzymatic scavenging system is the core pathway for ^1^O_2_ scavenging. It relies on naturally occurring substances in the organism to directly scavenge ^1^O_2_ through either physical quenching or chemical quenching [75].

### 4.1. Carotenoid-Mediated Protection Against Singlet Oxygen

Lipid-soluble carotenoids are regarded as the first line of defense for plants against ^1^O_2_ toxicity and one of the most important ^1^O_2_ quenchers in chloroplasts [76]. A variety of natural carotenoids exist in nature, all of which contain numerous conjugated double bonds. Carotenoids absorb energy from ^3^Chl* or ^1^O_2_ to transition from the “ground state” to the “excited state”; excited-state carotenoids then release energy through non-radiative transitions (e.g., heat dissipation) and return to the ground state. This recyclable property of carotenoids is the key to their “high efficiency” [77]. Under photoinhibitory conditions, the photoprotective effect of carotenoids can effectively quench ^1^O_2_ and reduce damage caused by ^1^O_2_.

Geranylgeranyl diphosphate (GGPP)—the direct precursor of carotenoid biosynthesis—forms phytoene under the action of phytoene synthase (PSY). Subsequently, other types of carotenoids are produced through reactions such as dehydrogenation and cyclization [78,79]. Among these, lycopene is a critical branch point in the carotenoid biosynthetic pathway: it is converted into substances such as β-carotene, lutein, zeaxanthin (Zx), and violaxan (Vx) thin via the action of a series of enzymes, including lycopene ε-cyclase (LCYe) and lycopene β-cyclase (LCYb). In the *szl1* mutant of *Arabidopsis thaliana*, reduced LCYb activity leads to a significant decrease in β-carotene content [80,81]. Under low-temperature and high-light conditions, *szl1* exhibits greater sensitivity of PSI to photooxidative stress, with ^1^O_2_-induced damage increasing in parallel with PSI damage. The fluorescence intensity of SOSGs in *szl1* is more than twice that of the wild type, and lipid peroxidation levels are significantly elevated. After isolating the PSI-LHCI complexes from *szl1* and the wild type, it was found that PSI in *szl1* is the main source of ^1^O_2_ production, while the ^1^O_2_ yield in PSII complexes is lower than that in PSI-LHCI complexes. This indicates that β-carotene plays a crucial role in photoprotection [20].

Genetic manipulation of carotenoid biosynthesis further supports the photoprotective functions of carotenoids. Overexpression of *AtPSY* in callus of *Arabidopsis* significantly increases the total carotenoid content, with β-carotene content exceeding that of lutein to become the main accumulated carotenoid [82]. LCYe is the first enzyme in the α-carotene biosynthetic branch; overexpression of *CrLCYe* in *Chlamydomonas* enhances the conversion of lycopene to α-carotene, thereby increasing lutein content [83]. Overexpression of *IbLCYB2* in sweet potato (*Ipomoea batatas* [L.] Lam.) significantly increases the contents of α-carotene, β-carotene, lutein, and Zx in transgenic sweet potato, enhancing tolerance to salt, drought, and oxidative stress [84].

qE (energy-dependent quenching) is a key component of NPQ, primarily induced by low pH in the thylakoid lumen. Zx is considered a critical cofactor and efficient executor of qE [85,86]. In the *npq4* of *Arabidopsis*, inactivation of *AtPsbS* prevents rapid induction of NPQ, indicating that PsbS is the core of rapid NPQ activation—and qE cannot form at all in *npq4* [87,88].

In the *npq1* of *Arabidopsis*, inactivation of *AtVDE* (violaxanthin de-epoxidase) prevents the conversion of Vx to Zx [89]; in contrast, the *lut2* of *Arabidopsis* cannot synthesize lutein or α-carotene due to *AtLCYe* inactivation [90]. A common feature of *npq1* and *lut2* is reduced qE levels, and the *npq1/lut2* double mutant completely lacks qE [89]. This suggests that in addition to Zx, lutein may also play a direct role in qE formation [91].

A recent study developed a novel multivariate workflow for NPQ induction analysis. By detecting NPQ induction data of *npq1*, *npq2* (Zx-enriched), wild type, and *npq4* after light and dark adaptation, the analysis revealed a synergistic interaction between Zx and PsbS: changes in Zx levels lead to alterations in PsbS components, and the presence of Zx can drive the enhancement of PsbS-dependent quenching, while the presence of PsbS also enables Zx to fully participate in NPQ [92]. β-cyclocitral (β-CC) is produced by the oxidation of β-carotene by ^1^O_2_. The changes in gene expression triggered by β-CC are associated with increased tolerance of *Arabidopsis* leaves to photooxidative damage; moreover, β-CC induces the significant expression of all ^1^O_2_ gene markers, indicating that β-CC acts as an intermediate in the ^1^O_2_ signaling pathway [93].

### 4.2. Ascorbate-Mediated Protection Against Singlet Oxygen

Ascorbic acid (AsA) is an effective chemical quencher of ^1^O_2_. It gradually quenches highly reactive ^1^O_2_ through redox reactions, while being oxidized to monodehydroascorbate (MDHA) itself. MDHA can then be regenerated into AsA via the “AsA-glutathione (GSH) cycle” [94,95]. Dehydroascorbate reductase (DHAR) is responsible for linking AsA and GSH in the redox system. The activity of AtDHAR decreases by approximately 60% in *dhr3*. Crossing *dhr3* with the GSH-deficient mutant-*cad2* yields the double mutant *dhr3/cad2*, in which the Fv/Fm is significantly reduced—indicating that AtDHAR3 works synergistically with GSH to accumulate AsA [96]. Ascorbate peroxidase (APX) requires AsA as an electron donor to scavenge the toxicity of H_2_O_2_ and generate water and MDHA. In *Arabidopsis*, there are stromal AtsAPX and thylakoidal AttAPX. Among them, plants overexpressing tAPX exhibit stronger resistance to photooxidative stress induced by methyl viologen spraying [97].

### 4.3. Tocopherol-Mediated Protection Against Singlet Oxygen

The increased content of lipid-soluble tocopherols (vitamin E) is associated with high-light stress, low temperature, drought, and other conditions. Tocopherols can scavenge various ROS and free radicals, and quench ^1^O_2_ [98,99,100]. Under high-light stress, the level of tocopherols in *Arabidopsis* leaves increases by 18-fold [101]. *AtVTE1* and *AtVTE2* encode tocopherol cyclase and homogentisate phytyltransferase (HPT), respectively. The *vte1* accumulates 2,3-dimethyl-5-phytyl-1,4-benzoquinone (DMPBQ)—an intermediate in the tocopherol biosynthesis pathway—and still retains redox activity; it only exhibits faster lipid peroxidation than the wild type under extreme low-temperature and continuous high-light stress. In contrast, the *vte2* mutant lacks the intermediate DMPBQ, leading to severe growth defects during germination and a significant increase in lipid peroxide levels [102,103,104]. The *vte1* produces more ^1^O_2_ under high-light stress. However, overexpression of the *AtSPS1* in *vte1* induces a significant accumulation of total plastoquinone (PQ-9). Meanwhile, under high-light stress, the production of ^1^O_2_ is significantly reduced and the consumption of PQ-9 is significantly accelerated. These results indicate that PQ-9 in thylakoid membranes can function as a ^1^O_2_ scavenger [105].

## 5. Singlet Oxygen-Mediated Chloroplast Signaling Transduction

For decades, ROS have been regarded as “waste products” that are toxic to cells [106]. With the continuous deepening of research, people have gradually uncovered the mysteries of ROS—they are now recognized to play varying degrees of roles in regulating almost all aspects of cell function and are of great significance for maintaining the intracellular redox state. Notably, ^1^O_2_ plays a crucial role in regulating the RS pathway between chloroplasts and the nucleus [44,107]. Under normal growth conditions, plants rely on endogenous ^1^O_2_ sensors to respond to ^1^O_2_ signals. EX1 and β-carotene are considered ^1^O_2_ sensors in plant cells, functioning in the grana margin (GM) and grana core (GC) regions respectively. Compared with the GC, the GM is more susceptible to damage by ^1^O_2_ [45,74,108].

### 5.1. Singlet Oxygen Signaling Transduction at the Grana Margins

EX1 and EX2 constitute core components of the chloroplast ^1^O_2_ signaling pathway. EX1 is a key component in the ^1^O_2_ signaling pathway. EXECUTER2 (EX2) is a homologous protein of EX1, and both contain a singlet oxygen sensor (SOS) domain. Inactivation of EX1 and EX2 is sufficient to eliminate ^1^O_2_-induced stress responses in *Arabidopsis* [45,109]. When the previously mentioned *flu* and *flu/ex2* double mutant are transferred from dark conditions to long-day conditions, their growth ceases. In *flu*, the expression of 245 genes is upregulated, and in *flu/ex2* double mutants, the upregulation amplitude of most of these genes is higher than that in *flu*. In contrast, *flu/ex1* double mutants and *flu/ex1/ex2* triple mutants can grow normally under long-day conditions, and the number of upregulated genes in *flu/ex1* is significantly reduced. These results indicate that inactivation of EX1 cannot completely eliminate ^1^O_2_-induced changes in gene expression, and EX2 may act as a regulator to control EX1 activity during this process [43,109].

The activation of EX1-mediated signaling is closely associated with the ^1^O_2_-dependent turnover of EX1. Subsequent studies revealed that ^1^O_2_-mediated responses in *flu* seedlings are not directly triggered by ^1^O_2_ but strictly depend on EX1. EX1 is localized at the margins of thylakoid grana. With the initiation of ^1^O_2_-mediated signal transduction, the protein abundance of EX1 decreases rapidly, which is dependent on the ATP-dependent zinc metalloprotease-FtsH [110]. FtsH is mainly localized to thylakoid membranes in plant chloroplasts, with particular enrichment at grana margins and unstacked regions of thylakoid membranes [111,112]. Mutations in *AtFtsH2* and *AtFtsH5* lead to leaf chlorosis in *var2* and *var1*, as well as sensitivity to photoinhibition and low-temperature stress, though their phenotypes under high-temperature stress are similar to the wild type. In contrast, loss of function of *AtFtsH8* and *AtFtsH1* does not result in obvious phenotypes [112,113]. FtsH cleaves the D1 protein during the disassembly of damaged PSII and plays a crucial role in EX1-mediated ^1^O_2_ signal transduction [110,114,115,116].

^1^O_2_ promotes the degradation of EX1 through post-translational modification (PTM). Specifically, ^1^O_2_ can oxidize Trp643 in the SOS domain of the EX1 protein. Replacing Trp643 with an ^1^O_2_-insensitive amino acid or deleting the SOS domain can eliminate EX1-mediated signal transduction and the occurrence of programmed cell death (PCD). The oxidized EX1 protein becomes a target of FtsH2 and is rapidly degraded under light [108,117]. FtsH2 is essential for ^1^O_2_-triggered RS; in *var2/flu* seedlings of *Arabidopsis*, inactivation of *FtsH2* inhibits 85% of EX1-dependent singlet oxygen responsive genes (*SORGs*) [118].

EX2 modulates the intensity of the EX1-dependent signal. EX2 has been confirmed to play a regulatory role in the EX1-mediated signaling pathway. As a homologous protein of EX1, Trp530 in its SOS domain can also undergo ^1^O_2_-dependent oxidation. This oxidation reduces the oxidation level at the EX1-Trp643 site, thereby slowing down the FtsH2-mediated degradation rate of EX1 and attenuating ^1^O_2_ signaling [117]. The expression level of EX1-dependent *SORGs* in *ex2/flu* is higher than that in *flu*; however, overexpression of *EX2* in *ex2/flu* seedlings restores the expression level of these *SORGs* [109]. Although the EX1/EX2 pathway provides the best-characterized molecular framework for ^1^O_2_ signaling in higher plants, evidence from other oxygenic photosynthetic organisms suggests that the signaling function of ^1^O_2_ is more broadly conserved [119,120].

In *Arabidopsis*, an important question is how EX1-mediated ^1^O_2_ signaling is transmitted from the chloroplast to the nucleus. Mutations in the Genomes Uncoupled (GUN) genes of *Arabidopsis* result in poor chloroplast development and abnormal expression of some photosynthesis-associated nuclear genes (*PhANGs*) [121,122]. GUN4 and GUN5 physically interact with the N-terminal region of EX1. Treatment with 50 µM RB under illumination at 40 µmol·m^−2^·s^−1^ for 10–30 min generates ^1^O_2_, which attenuates this protein interaction. Transgenic seedlings expressing EX1-GFP were first dark-acclimated for 6.5 d and then exposed to light at 200 µmol·m^−2^·s^−1^ for 30 min. Confocal imaging revealed a gradual increase in EX1-GFP signal within the nucleus, accompanied by a mild reduction in the plastidic EX1-GFP signal. Inhibition of de novo protein synthesis with cycloheximide (CHX) did not alter the nuclear accumulation of EX1-GFP, demonstrating that ^1^O_2_ signaling initiates the translocation of EX1 from chloroplasts to the nucleus. After translocating to the nucleus, EX1 interacts with WRKY18 and WRKY40, and the presence of ^1^O_2_ promotes this interaction. Furthermore, EX1 has transcriptional activation activity, suggesting that it can act as a transcriptional co-regulator to jointly regulate the expression of *SORGs* with WRKY transcription factors [123].

**Figure 2:**
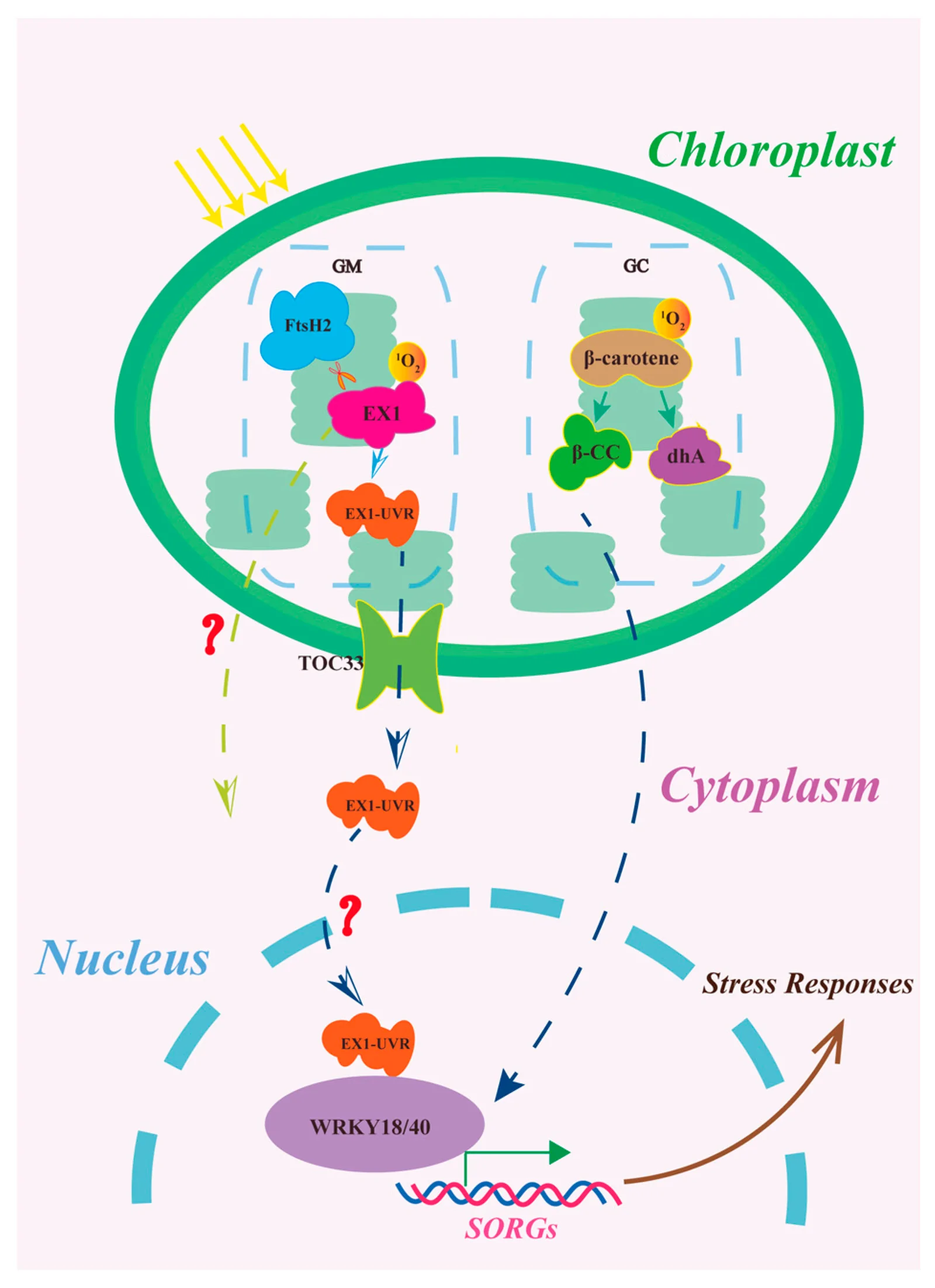
Schematic diagram of the EX1 signaling pathway. Under photooxidative stress, ^1^O_2_ is generated in chloroplast thylakoid membranes. At the GM, ^1^O_2_ promotes the oxidation-dependent modification and FtsH-mediated processing of EX1, which initiates EX1-dependent retrograde signaling. The resulting signal is transmitted from the chloroplast, possibly involving the outer envelope component TOC33, to the nucleus, where transcription factors such as WRKY18 and WRKY40 activate *SORGs* and downstream stress responses. In parallel, ^1^O_2_ oxidation of β-carotene in the GC produces β-CC and related apocarotenoid signals, including dhA, which also contribute to nuclear gene regulation. Question marks indicate steps that remain unresolved.

Recent findings involving TOC33 provide further mechanistic insight into the translocation and processing of EX1. TOC33, a protein in the chloroplast precursor protein transport machinery (TOC-TIC complex), plays a critical role in the translocation of EX1. Mutation of TOC33 can suppress the phenotype of *flu* mutants caused by ^1^O_2_ outburst but does not affect ^1^O_2_ production or the content and localization of EX1 in chloroplasts. After EX1 is cleaved by the FtsH2 protease, its UVR domain is released. TOC33 can interact with EX1 fragments containing either a transit peptide or the UVR domain. The UVR-GFP signal can accumulate in the nucleus, and only the UVR domain can interact with WRKY18/40 to regulate the expression of *SORGs*—other truncated EX1 fragments or the full-length EX1 protein cannot interact with WRKY18/40 (Figure 2) [124].

Downstream of the EX1/EX2–WRKY module, ABI4 further contributes to the transcriptional response. ABI4 is an important AP2/ERF family transcription factor in plants, playing key roles in plant growth, development, and stress responses. ABI4 is localized downstream of WRKY transcription factors and EX1/EX2; inactivation of ABI4 in *flu/abi4* of *Arabidopsis* rescues the phenotype of *flu* under light-dark cycles [125,126].

In parallel with the EX1-dependent pathway, SAFE1 provides an additional layer of protection and regulation. SAFE1 is localized to the chloroplast stroma and can protect GM from ^1^O_2_ damage. In *flu/ex1*, after ^1^O_2_ generation, the structure of GMs remains intact, the number and size of plastoglobules (PGs) associated with thylakoid membranes show no significant changes, and chloroplast function is preserved. In contrast, in the *flu/ex1/safe1* triple mutant, GM-specific proteins including POR, CURT1, CHLI, and CHLM undergo significant degradation, accompanied by massive aggregation of PGs and induction of cell death. Detection of the transcriptional levels of *SORGs* in *flu/ex1/safe1* shows that loss of SAFE1 restores ^1^O_2_-induced transcriptional changes to the level of *flu*, or even higher. This indicates that SAFE1 can weaken stress responses by inhibiting ^1^O_2_-induced downstream changes in gene expression. Meanwhile, there is no significant difference in the efficiency of EX1-mediated signal transmission between *flu/safe1* and *flu*, and key steps of the EX1 pathway (such as EX1 oxidation and FtsH2 cleavage) are not affected. These results suggest that SAFE1 acts in an EX1-independent ^1^O_2_ signaling pathway and cooperates with the EX1 pathway to achieve precise regulation of ^1^O_2_ signaling [74].

Collectively, accumulated studies have established that EX1 acts as the central sensor and signaling component in the ^1^O_2_ signaling pathway, while EX2 modulates the functional activity of EX1. FtsH2 mediates the proteolytic cleavage of EX1 to generate signal-competent EX1 fragments, and TOC33 facilitates signal communication between these fragments and nuclear transcriptional regulators. ABI4 functions downstream of this chloroplast retrograde signaling pathway. Meanwhile, SAFE1 operates through a parallel EX1-independent pathway to cooperatively regulate cellular responses together with EX1.

### 5.2. Singlet Oxygen Signaling Transduction at the Grana Cores

In the GC region, the generated ^1^O_2_ is mainly scavenged by β-carotene and α-tocopherol [127,128]. The ^1^O_2_ signaling pathway is primarily mediated by the OXI1 kinase and β-CC and mainly responds to ^1^O_2_ produced by PSII under stress conditions. As previously mentioned, ^1^O_2_ production is induced under high-light inhibition. Studies on the *ch1* have revealed that ^1^O_2_-induced changes in gene expression can trigger PCD, and the OXI1 is thought to play a key role in this process [129]. *OXI1* encodes an AGC kinase, which is closely associated with ROS and plant-pathogen interactions [130,131,132]. Inactivation of the OXI1 protein enhances resistance to high-light stress, whereas *OXI1*-overexpressing lines exhibit accelerated senescence and PCD under high-light stress. Further research has shown that ^1^O_2_-induced PCD in *ch1* is related to jasmonic acid (JA) biosynthesis. In the *oxi1*, the expression of genes in the JA pathway is downregulated, JA levels are reduced, and the synthesis of salicylic acid (SA) is almost completely blocked. DAD1 and DAD2, together with OXI1, play roles in regulating JA synthesis and PCD processes, and DAD genes negatively regulate the expression of *OXI1* [133,134].

Oxidative cleavage of the double bonds at positions 7 and 8 in the polyene chain of β-carotene by ^1^O_2_ produces β-CC; if the double bonds at positions 9 and 10 are oxidatively cleaved, β-ionone (β-I) is generated. β-I is further oxidized by ^1^O_2_ to eventually form dihydroactinidiolide (dhA) [93,135]. Under high-light stress, β-CC, β-I, and dhA accumulate rapidly. Among them, β-CC and dhA are lipid-soluble small-molecule organic compounds that can cross membranes without the assistance of transporters. Thus, they can act as signaling molecules to transmit signals between different organelles—this forms the theoretical basis for β-carotene being regarded as an ^1^O_2_ sensor. In contrast, β-I has not been found to be involved in signal transmission [93,136]. In addition to being generated by ^1^O_2_ oxidation, β-I can also be produced through enzymatic reactions mediated by carotenoid cleavage dioxygenases (CCDs) [137]. Theoretically, CCDs may also be involved in β-CC production; however, in *Arabidopsis*, these CCDs are unlikely to participate in β-CC production in leaves [93,138]. *Arabidopsis* contains 9 CCD members, which are mainly divided into two subfamilies: CCDs and 9-cis-epoxycarotenoid dioxygenases (NCEDs). Among the CCD subfamily, members CCD1, CCD4, and CCD7 preferentially mediate β-I production [139]. Under high-light stress, these CCD genes are not induced—CCD4 is even repressed—and the accumulation of β-CC and β-I in *Arabidopsis* mutants with inactivated CCDs is not inhibited. This indicates that these genes are not involved in regulating stress responses under light stress [93,138].

Volatile oxidative derivatives of β-carotene in *Arabidopsis* leaves, generated under high-light stress, also include α,β-unsaturated carbonyls; such metabolites are classified as reactive electrophilic species (RES) [140]. RES exhibit high reactivity, so excessive RES can cause cell damage, while low levels of RES can participate in plant stress responses, regulate cellular gene expression, and support cell survival under stress conditions [141]. β-CC and dhA can induce the expression of *SORGs* [93,135]. *Arabidopsis* leaves were treated with different concentrations of β-CC and β-I and found that β-CC significantly induced the expression of several selected *SORGs* in a clear dose-dependent manner. In contrast, β-I only slightly induced *AtCLO3*. Transcriptome analysis showed that after β-CC treatment, the expression of genes related to JA synthesis, ethylene synthesis, and various defense pathways in *Arabidopsis* was upregulated—exhibiting similar gene expression patterns to the *flu* [93]. The expression level of *SORGs* in *Arabidopsis* also showed a clear dose dependence on dhA; conversely, *AtCAO1* was significantly repressed. Moreover, dhA treatment failed to induce the expression of *AtAPX1* and the H_2_O_2_-responsive gene *AtCAT2* [135]. These results are similar to those of β-CC treatment, indicating that β-CC and dhA have a significant correlation with ^1^O_2_ induction (Figure 2).

^1^O_2_-induced genetic changes can trigger PCD or enhance plant tolerance to photooxidative stress [133]. The PCD process is regulated by proteins such as EX1, OXI1, ACD2, and PUB4 [123,134,142,143], whereas gene expression induced by β-CC and dhA signaling does not lead to cell degradation. *Arabidopsis* plants treated with β-CC or dhA exhibit greater tolerance to high-light stress compared to untreated plants, with reduced accumulation of lipid peroxides [93,135]. Lipid peroxides are not only hallmark products of photooxidation, but their decomposition-derived reactive carbonyl species (RCS) can directly exacerbate photooxidative toxicity [144,145]. Notably, β-CC enhances the detoxification capacity of the xenobiotic detoxification system regulated by SCARECROW-LIKE14 (SCL14) toward RCS [136]. These findings indicate that β-CC and dhA can induce plant adaptation to high-light stress, and β-CC may act as an “endogenous safener” involved in plant acclimation processes.

## 6. Future Perspectives

Although substantial progress has been made in ^1^O_2_ signaling in plants, many issues remain to be addressed. For example, although the importance of EX1 in signal transduction has been confirmed, the mechanism by which EX1 translocates from chloroplasts to the nucleus is not fully understood, and it is unclear whether EX1 cooperates with other nuclear transcription factors to regulate the expression of *PhANGs*. In addition, a series of pathways require further investigation, such as whether there are other EX1-independent ^1^O_2_ signaling pathways (similar to the SAFE1-mediated pathway) involved in ^1^O_2_ signal regulation and whether other types of carotenoid derivatives participate in such regulation.

In the future, we can clarify the specific function of each component by further analyzing the existing ^1^O_2_ signaling pathways, continue to explore new ^1^O_2_ signaling components, and identify more genes and proteins involved in signal transduction. In natural environments, plants frequently encounter combined abiotic stresses such as high temperature plus high light, or high temperature plus drought. The crosstalk mechanisms linking ^1^O_2_ signaling with plant hormone pathways and other environmental stress pathways remain to be further investigated. Future research should focus on dissecting the translocation mechanism of EX1 protein, identifying novel ^1^O_2_ sensors and signaling components, and clarifying how signal intensity, duration and subcellular localization govern the balance between PCD and stress acclimation.

## 7. Conclusions

^1^O_2_ is a unique ROS continuously produced in plant leaves under light, mainly when chlorophylls transfer excitation energy to molecular oxygen. The PSII reaction center and LHCs are the primary sites of ^1^O_2_ production, making PSII a main target of ^1^O_2_ attack. A clear correlation exists between the rate of ^1^O_2_ formation and the rate of PSII photo-damage. Evidence from the *Arabidopsis flu/ex1/safe1* model suggests that grana margins are particularly sensitive sites of ^1^O_2_-induced damage. In this system, enhanced ^1^O_2_ production is associated with more severe GM damage, cell death, and PSII impairment. However, the available data do not establish a precise quantitative threshold separating ^1^O_2_ signaling from toxicity, nor do they fully resolve the compartment-specific or temporal dynamics of this transition. Therefore, the potential interaction between ^1^O_2_ signaling and other plant stress or hormone signals should be interpreted as context-dependent and remains an important topic for future investigation.

To avoid oxidative damage triggered by ^1^O_2_, plants have evolved various coping mechanisms. Lipid-soluble carotenoids are considered the first line of defense against ^1^O_2_ toxicity in plants, whereas ascorbate, tocopherols, plastoquinone/plastoquinol, plastochromanol, and glutathione contribute to ^1^O_2_ quenching, lipid-radical scavenging, antioxidant regeneration, and redox buffering. Thus, these metabolites should not be considered independent or quantitatively equivalent ^1^O_2_ scavengers. Instead, their functions are integrated to restrict oxidative damage while maintaining the redox conditions required for signaling.

The available evidence supports the existence of spatially distinct ^1^O_2_ signaling pathways in chloroplasts. ^1^O_2_ signaling in the GM region is mainly mediated by the EX1 and, independently, by SAFE1. The EX1 pathway involves the oxidation and cleavage of EX1, the interaction between the EX1-UVR domain and TOC33, and the nuclear localization of EX1. In the nucleus, the EX1-UVR fragment cooperates with members of the WRKY transcription factor family to regulate the expression of *SORGs*. The SAFE1 pathway provides an additional route for ^1^O_2_-dependent transcriptional regulation. In the *flu/ex1* mutant, inactivation of SAFE1 restores ^1^O_2_-induced transcriptional changes to the level of the *flu* mutant, or even higher. In the GC region, ^1^O_2_ signaling is mainly mediated by the OXI1 kinase and β-carotene and primarily responds to ^1^O_2_ produced by PSII under stress conditions. These findings indicate that the biological outcome of ^1^O_2_ production is determined not only by its abundance but also by its site of production and the signaling components present in that compartment.

Carotenoid-derived molecules provide another layer of ^1^O_2_-dependent retrograde signaling. β-carotene oxidation products, including β-CC and dhA, can induce several ^1^O_2_-responsive marker genes and transmit information between chloroplasts and the nucleus. Although β-CC and dhA induce several ^1^O_2_-responsive marker genes that are also regulated in ^1^O_2_-producing mutants, this should not be interpreted as activation of an identical downstream program. ^1^O_2_ signaling is context-dependent and may lead either to EX1/2-dependent programmed cell death or to acclimation. In *flu*, direct chloroplast ^1^O_2_ production can trigger cell-death-related signaling, whereas β-CC and dhA are carotenoid-derived oxidation products formed downstream of ^1^O_2_ quenching and appear to function mainly as retrograde signals for stress acclimation. Consistently, β-CC and dhA treatments reduce lipid peroxidation and PSII photoinhibition under high-light stress. For β-CC, this protective effect is associated with SA accumulation, NPR1 nuclear localization, and GST-mediated detoxification responses. Therefore, the distinct downstream effects likely reflect differences in signal intensity, timing, subcellular origin, and pathway branching rather than a contradiction in the transcriptional data.

## Data Availability

No new data were created or analyzed in this study. Data sharing is not applicable to this article.

## Abbreviations

The following abbreviations are used in this manuscript:

RS	retrograde signaling
SO	singlet oxygen
ROS	reactive oxygen species
NPQ	non-photochemical quenching
LHCII	light-harvesting complex II
PSII	photosystem II
PSI	photosystem I
CAO	Chlorophyll a oxygenase
ISC	intersystem crossing
Pheo	pheophytin
PQ	plastoquinone
OEC	oxygen-evolving complex
Proto IX	protoporphyrin IX
Pchlide	protochlorophyllide
EX1	EXECUTER1
ALA	5-aminolevulinic acid
SOSG	singlet oxygen sensor green
PEG	polyethylene glycol
OPTM	oxidative post-translational modifications
Cys	cysteine
Met	methionine
Tyr	tyrosine
Phe	phenylalanine
Trp	tryptophan
OIA	oxindolylalanine
NFK	N-formylkynurenine
KYN	kynurenine
EF-G	elongation factor G
MDA	malondialdehyde
POR	protochlorophyll oxidoreductase
CHLI	Mg^2+^ chelatase subunit I
CHLM	Mg^2+^ protoporphyrin IX methyltransferase
RB	rose bengal
GGPP	Geranylgeranyl diphosphate
PSY	phytoene synthase
Zx	zeaxanthin
Vx	violaxanthin
LCYe	lycopene ε-cyclase
LCYb	lycopene β-cyclase
qE	energy-dependent quenching
VDE	violaxanthin de-epoxidase
β-CC	β-cyclocitral
AsA	Ascorbic acid
MDHA	monodehydroascorbate
GSH	glutathione
DHAR	Dehydroascorbate reductase
Fv/Fm	maximum quantum yield of PSII
APX	Ascorbate peroxidase
sAPX	stromal ascorbate peroxidase
tAPX	thylakoidal ascorbate peroxidase
HPT	homogentisate phytyltransferase
DMPBQ	2,3-dimethyl-5-phytyl-1,4-benzoquinone
PQ-9	plastoquinone-9
GM	grana margin
GC	grana core
EX2	EXECUTER2
SOS	Singlet Oxygen Sensor
FtsH	ATP-dependent zinc metalloprotease
PTM	Post-Translational Modification
PCD	programmed cell death
SORGs	single oxygen responsive genes
GUN	genomes uncoupled
PhANGs	photosynthesis-associated nuclear genes
CHX	cycloheximide
TOC-TIC	translocon at the outer/inner envelope membrane of chloroplasts
PGs	plastoglobules
ABI4	ABA INSENSITIVE 4
JA	jasmonic acid
SA	salicylic acid
β-I	β-ionone
dhA	dihydroactinidiolide
CCDs	carotenoid cleavage dioxygenases
NCEDs	9-cis-epoxycarotenoid dioxygenases
RES	reactive electrophilic species
RCS	reactive carbonyl species
SCL14	SCARECROW-LIKE14
